# Comparative Analysis of P63, Maspin and Matrix Metalloproteinase 2 Expression in Mucoepidermoid Carcinoma and Adenoid Cystic Carcinoma of Salivary Glands

**DOI:** 10.30476/DENTJODS.2019.77868.0

**Published:** 2020-06

**Authors:** Nasim Taghavi, Farzad Yazdani, Alireza Akbarzadeh Baghban, Soudabeh Sargolzaei, Parisa Kardouni Khoozestani

**Affiliations:** 1 Dept. of Oral & Maxillofacial Pathology, Shahid Beheshti University of Medical Sciences, Tehran, Iran; 2 Dept. of Pathology, School of Medicine, Tehran University of Medical Sciences, Tehran, Iran; 3 Proteomics Research Center, Dept. of Basic Science, School of Rehabilitation, Shahid Beheshti University of Medical Sciences, Tehran, Iran; 4 Dept. of Oral and Maxillofacial Pathology, Dental School, Anzali International Campus, Guilan university of Medical Sciences, Rasht, Iran

**Keywords:** Mucoepidermoid Carcinoma, Adenoid Cystic Carcinoma, P63, Maspin, Matrix Metalloproteinase

## Abstract

**Statement of the Problem:**

The tumor suppressor role of myoepithelial cells and related mechanisms in breast tumors are well understood. However, the role of these cells in tumors of salivary glands is debatable.

**Purpose:**

The present study was designed to determine the expression of p63, mammary serine protease inhibitor (maspin) and matrix metalloproteinase 2 (MMP-2) in mucoepidermoid carcinoma (MEC) and adenoid cystic carcinoma (ADCC) of salivary glands due to various cellular differentiation and structure. The association between the expression of these markers and clinicopathologic features and myoepithelial differentiation were also evaluated.

**Materials and Method:**

P63, maspin, and MMP-2 expression were immunohistochemically studied in 67 cases including35 cases of MEC and 32 cases of ADCC. The smooth muscle actin (SMA) staining was also applied to confirm the presence of myoepithelial differentiation. Data was analyzed using Chi-square test, Mann-Whitney U test and t-test.

**Results:**

The expression of p63 (*p*= 0.009) and maspin (*p*= 0.012) significantly differed between the study groups. P63 positive cells in MEC were negative for SMA staining in contrast to ADCC. Furthermore, the expression of P63 (*p*= 0.045) and maspin (*p*= 0.019) significantly and inversely correlated with histologic grade in ADCC. Likewise, positive significant correlation was detected between histologic grade and expression of P63 (*p*= 0.018) and MMP-2 (*p*= 0.003) in MEC samples.

**Conclusion:**

Our findings showed that MEC is devoid of myoepithelial cells. The difference in expression of P63 and maspin in ADCC and MEC highlighted the role and presence of myoepithelial cells in ADCC. Indeed, the high expression of P63 and maspin in well-differentiated ADCCs suggests the tumor suppressor effect of myoepithelial cells. Considering the association between the evaluated markers and histological grade, p63 in both tumors, maspin in ADCC and MMP-2 in MEC may be efficient predictors of clinical behavior.

## Introduction

The myoepithelial and luminal epithelial cells are two distinct cellular lineages in the basic epithelial structure of salivary glands and the origins of most salivary gland tumors [ 1 ]. Regardless of cell of origin, cellular differentiation in salivary glands tumors specifies the final histologic features of tumors. Accordingly, myoepithelial cells are key cellular participants during tumorigenesis of salivary glands [ 2 - 3 ].

Mucoepidermoid carcinoma (MEC) is the most common malignant salivary glands tumor with variable potentials for invasion [ 4 - 5 ]. Histologically, MEC is characterized with the presence of squamous, intermediate and mucous cells. Myoepithelial differentiation in MEC is a matter of controversy. The investigation of Regezi *et al*. [ 6 ] revealed that myoepithelial cells were rare in MEC. Contradictory data has been documented by ultrastructural and immunohistochemical studies [ 7 ]. 

Adenoid cystic carcinoma (ADCC) is the second most common malignancy of salivary glands characterized by persistent growth, perineural invasion and distant metastasis. ADCC displays a dual differentiation of myoepithelial and epithelial cells which is evident in three histopathologic patterns including cribriform, tubular and solid [ 4 , 8 ]. Evidence shows that cribriform and tubular pattern have a more favorable prognosis than solid pattern [ 9 ].

P63 protein, a homologue of P53, is required for epidermal morphogenesis. It is also a sensitive marker for neoplastic myoepithelial cells [ 1 - 3 ]. P63 overexpression has been reported in oral squamous cell carcinoma and urothelial carcinoma [ 10 - 11 ]. Moreover, Fonseca *et al*. [ 5 ] recommended P63 as a helpful marker for distinguishing low grade MEC from papillary cystadenoma. Nevertheless, the studies focusing on the prognostic value of P63 in salivary gland tumors are limited.

Mammary serine protease inhibitor (maspin) is a marker of the serine protease inhibitors family. Maspin suppresses tumor cell migration, angiogenesis, and metastatic spread, giving a peculiar tumor suppressive function on this protein. It was initially distinguished in myoepithelial cells of human breast and demonstrates alterable expression in various types of cancer [ 12 - 13 ]. A recent study demonstrated the down regulation of maspin in epithelial component of ex-pleomorphic adenoma during malignant transformation despite maspin upregulation in transformed myoepithelial cells [ 14 ]. Matrix metalloproteinases (MMPs) are a family of zinc dependent endopeptidases with enzymatic activity against basement membrane (BM) and extracellular matrix (ECM) proteins. Some of MMPs including matrix metalloproteinase 2(MMP-2) and matrix metalloproteinase 9(MMP-9) have significant roles in different stages of tumor progression [ 15 - 16 ]. The prognostic value of special MMPs has been demonstrated in malignant tumors such as oral squamous cell carcinoma (OSCC) [ 17 ]. Furthermore, studies have proposed the modulatory effects of myoepithelial cells on MMPs produced by fibroblasts and cancer cells [ 2 , 18 ]. 

Accordingly, the aim of the present study was to determine and compare the expression of P63, maspin and MMP-2 in MEC and ADCC, two common salivary gland tumors with various cellular differentiation and structures. We also investigated the association of markers’ expression with clinicopathologic features and presence of myoepithelial cells in both tumors.

## Materials and Method

In total, 35 cases of MEC and 32 cases of ADCC of salivary glands were retrieved from the archives of the Department of Oral Pathology, University of Medical Sciences, Tehran, Iran. After reviewing patients’ file, all slides were re-evaluated to confirm the diagnosis according to WHO classification [ 19 ]. The histological grading of MECs and ADCCs was conducted according to the Brandwein system [ 20 ] and WHO classification [ 19 ], respectively. The study was approved by the Research Ethical Committee of related University of Medical Sciences and performed in accordance with the *declaration of Helsinki* (Code: 95-1208).

For immunohistochemistry staining, 3-µm sections of routinely processed paraffin embedded blocks were dewaxed with xylene and then hydrated in graded ethanol for antigen retrieval. The slides were immersed and heated in 10 mm/L citrate buffer (pH 0.6) in microwave oven. After cooling to room temperature, the slides were incubated with primary antibodies against p63 (ready to use, Dako, Denmark), maspin (1:50, Novocastra, UK) and MMP-2(1:60, Novocastra, UK). To identify p63 positive cells as myoepithelial cells, immunoexpression of smooth muscle actin (SMA) (Novocastra, UK) was assessed in all the specimens. All slides were subsequently exposed to Dako Envision ^TM^, diaminobenzidine (DAB; DAKO) and counterstained with Mayer’s hematoxylin. OSCC, ulcerative colitis, normal salivary gland tissue and bowel wall were used as positive control for p63, maspin, MMP-2 and SMA, respectively. Negative controls were obtained using non-immune serum in TBS instead of primary antibody.

P63 nuclear immunostaining was scored as follows: negative; less than 10% of tumor cells stained, weakly positive; 10-25% of tumor cells stained, moderately positive; 26-75% of tumor cells stained, and strongly positive; 76-100% of tumor cells stained [ 21 ].

Maspin nuclear, cytoplasmic or nuclear-cytoplasmic immunoreaction was categorized into three groups based on the percentage of the positive tumor cells as low (up to 20% tumor cells stained), intermediate (20-49% of tumor cells stained), and high (≥50% of tumor cells stained) [ 12 ].

MMP-2 cytoplasmic expression was assessed using a semi quantitative scoring system based on proportion and intensity of staining. The percentage of positive cells was scored as 0 (negative), 1 (<10% of tumor cells stained), 2 (10-50% of tumor cells stained), and 3(>50% of tumor cells stained). The intensities were scored as 0 (no staining), 1(weak staining), 2(moderate staining) and 3(strong staining). Finally, the two scores were multiplied, providing the final scores as 0-1 (-), 2-3 (+), and ≥4 (++) [ 15 ]. All slides were evaluated by two pathologists without knowledge of the clinical outcome.

Data analysis was carried out in SPSS 18 software (SPSS, Inc, Chicago, IL, USA). Mann-Whitney U test, chi - square test, and independent t-test were applied to compare the expression of P63, maspin and MMP-2 between MEC and ADCC and to ascertain any association between markers expression and clinicopathologic characteristics. 

The Spearman’s correlation coefficient was used to analyze the co-expression of P63, maspin and MMP-2. In this study, *p*< 0.05 was considered statistically significant.

## Results

Sixty-seven cases were included in the present study, comprising 35 cases of MEC and 32 cases of ADCC. Clinical, demographic and pathologic characteristics of the samples are shown in Table 1.

**Table 1:** Clinicopathologic features of mucoepidermoid carcinoma and adenoid cystic carcinoma patients

	Mucoepidermoidcarcinoma	Adenoid cystic carcinoma
Characteristic	n (%)	n (%)
NO of patients	35(100)	32(100)
Age (years)		
Mean (SD)	42.17(17.13)	49.96(13.17)
<40	19(54.2%)	9(28.1%)
≥40	16(45.8%)	23(71.9%)
Gender		
Female	22(62.9%)	20(62.5%)
Male	13(37.1%)	12(37.5%)
Location		
Major salivary gland	17(48.6%)	6(18.75%)
Minor salivary gland	18(51.4%)	26(81.25%)
Size (cm)		
<4	25(71%)	16(50%)
≥4	10(29%)	16(50%)
Grade		
I (low)	14(40%)	10(31.2%)
II (intermediate)	6(17.1%)	15(46.8%)
III (high)	15(42.8%)	7(21.8%)
Neural invasion		
No	32(91.4%)	14(56.2%)
Yes	3(8.6%)	18(43.7%)
Lymph node metastasis		
No	34(97.1%)	28(87.5%)
Yes	1(2.9%)	4(12.5%)

**Figure 1:**
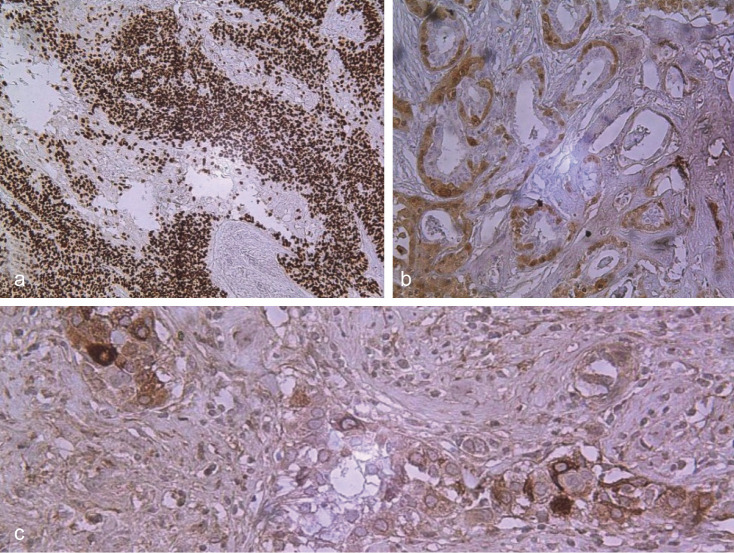
**a:** P63 nuclear expression in MEC(x100), **b:** Maspin nuclear-cytoplasmic expression in MEC(x200), **c:** MMP-2 cytoplasmic expression in MEC(x200)

P63 immunoreactivity was detected in all MEC samples mainly in epidermoid cells and occasionally in intermediate cells as nuclear staining (Figure 1a). In addition, the epidermoid and intermediate cells revealed cytoplasmic or nuclear-cytoplasmic expression of maspin in 31(88.6%) cases of MEC (Figure 1b). The cytoplasmic expression of MMP-2 was further found in epidermoid cells, intermediate cells and tumor stroma of 26(72.4%) MEC samples (Figure 1c). SMA staining was negative for all P63 positive cells in MEC samples.

**Figure 2:**
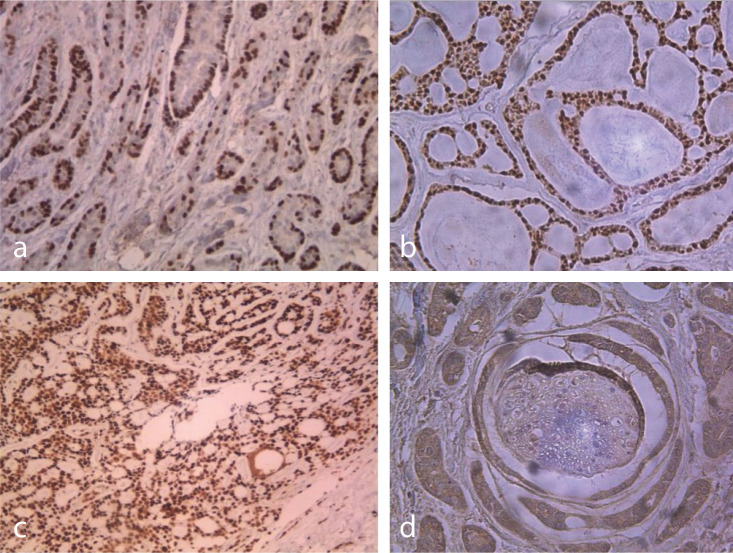
**a and b:** p63 nuclear expression in ADCC (x200), **c:** Maspin nuclear expression in ADCC(x200), **d:** MMP-2 cytoplasmic expression in ADCC(x 400)

In ADCC specimens, 31 (96.8%) cases showed P63 positivity in abluminal cells (Figure 2a, 2b). Maspin and MMP-2 expression were observed in 29 (90.7%) and 30 (93.7%) cases, respectively. Luminal and abluminal cells demonstrated nuclear expression of maspin while cytoplasmic staining of MMP-2 (Figure 2c, 2d). SMA immunoreaction was detected in all P63 positive cells confirming the differentiation of myoepithelial cells in ADCC.

Overall, significant higher expression of p63 (*p*= 0.009) and maspin (*p*= 0.012) were observed in ADCC than MEC samples. The associations of P63, maspin, and MMP-2 expression with clinicopathologic features of tumors are summarized in Table 2.

**Table 2:** *p* Values for evaluating association of P63, maspin and MMP-2 expression with clinicopathologic features of mucoepidermoid carcinoma and adenoid cystic carcinoma

Tumor/characteristic	P63 expression	Maspinexpression	MMP-2 expression
Mucoepidermoid carcinoma			
Age group	0.109^a^	0.567^a^	0.832^a^
Sex	0.601^a^	0.428^a^	0.960^a^
Tumor size	0.122^a^	0.900^a^	0.602^a^
Histologic grade	0.018^b^	0.133^a^	0.003^b^
Perineural invasion	0.934^a^	0.556^a^	0.411^a^
Lymph node metastasis	0.629^a^	0.914^a^	0.800^a^
Adenoid cystic carcinoma			
Age group	0.773^a^	0.409^a^	0.341^a^
Sex	0.182^a^	0.224^a^	0.687^a^
Tumor size	0.361^a^	0.239^a^	0.539^a^
Histologic grade	0.045^b^	0.019^b^	0.906^a^
Perineural invasion	0.464^a^	0.649^a^	0.206^a^
Lymph node metastasis	0.805^a^	1.00^a^	0.457^a^

a Based on Mann-Whitney test

b Based on Chi-square test

Bold values are statistically significant (p< 0.05)

In ADCC, the expression of P63 (*p*= 0.045) and maspin (*p*= 0.019) inversely correlated with histologic grade. On the other hand, histologic grade in MEC significantly correlated with the expression of P63 (*p*= 0.018) and MMP-2 (*p*= 0.003). Besides, t-test showed significant correlation between larger tumor size and lymph node metastasis in ADCC (*p*= 0.016). Spearman’s rank correlation coefficient revealed a significant correlation between P63 and maspin expression in ADCC (r= 0.588, *p*< 0.001) and the expression of P63 and MMP-2 in MEC (r= 0.360, *p*= 0.033). 

## Discussion

In the present study, immunohistochemical expression of P63, maspin and MMP-2 were assessed in MEC and ADCC, two most common malignant salivary glands tumors with various cellular differentiations. In MEC, the P63 expression was observed mainly in epidermoid cells and scattered in intermediate cells. The P63 stained cells were unreactive for SMA indicating the absence of myoepithelial cells in MEC.

This finding of ours is supported by some previously reported data [ 2 , 22 ]. On the other hand, abluminal p63 positive cells in ADCC were reactive for SMA confirming participation of myoepithelial cells in ADCC which was in line with Prasad *et al*. [ 23 ] and Savera *et al*. [ 2 ] studies. Myoepithelial cells are critical components of some salivary glands and breast tumors. They function as tumor suppressor by secreting large amount of angiogenesis inhibitors, protease inhibitors (Maspin, PAI-1), ECM proteins, tissue inhibitor metalloproteinase-1 (TIMP-1) and synthesis of BM [ 2 , 3 , 18 ]. 

In our study, a significantly higher expression of p63 was found in ADCC compared with MEC samples. We also demonstrated a significantly higher p63 expression in low grade ADCC compared with high grade cases of MEC. These differences may be partly due to various tumor cells differentiation and also to the presence of myoepithelial cells in ADCC. In MECs, p63 expression was prominently detected in epidermoid cells which were numerous in high grade tumors. This is while the main p63 positive cells in ADCC were myoepithelial cells surrounding the tubular and cribriform structures (Grade I, II). Furthermore, the presence of few myoepithelial cells in solid type (Grade III) which sparsely expressed p63 supports the tumor suppressor effect of these cells. In line with the current study, Ramer *et al*. [ 8 ] suggested the cutoff of 35% positivity for p63 as a prognostic indicator in ADCC. There are also reports emphasizing the correlation of p63 expression with histologic grade and prognosis in OSCC and meningioma [ 10 , 24 ].

A number of studies have detected maspin immunoreaction in different cells of epithelial origin especially in myoepithelial cells of mammary and salivary glands and arising tumors. Evidence implicates that neoplasms expressing high level of maspin have more favorite biologic behavior [ 25 ]. Navaro *et al*. [ 26 ] studied maspin expression in normal and neoplastic salivary glands tumors including pleomorphic adenoma, epithelial-myoepithelial carcinoma, and adenoid cystic carcinoma. They reported down regulation of maspin in tumors with more advanced histologic grade. Our findings demonstrated a statistically significant difference in the expression of maspin between two groups. Further analysis revealed higher maspin expression in well-differentiated ADCCs but there was no association between clinicopathologic features and maspin expression in MEC. These differences may be related to the myoepithelial differentiation in ADCC and to the variable cellular localization of maspin in these tumors. According to existing data, maspin expression is gradually lost in tumors in which only epithelial cells undergo malignant transformation. In malignant tumors containing myoepithelial cells; however, maspin is highly and consecutively upregulated [ 12 ]. In addition, evidence showed that nuclear localization of maspin is reflective of a less aggressive behavior. On the other hand, tumors with cytoplasmic or mixed nuclear-cytoplasmic expression of maspin show more aggressive behavior suggesting a crucial tumor suppressive effect for nuclear expressed maspin [ 25 ]. In the present study, maspin showed nuclear staining in ADCC while cytoplasmic or nuclear-cytoplasmic staining in MEC samples. Of interest, we found a significant association between maspin and P63 expression in ADCC. This finding was supported by a previous study on breast cancers, indicating maspin and P63 as the most promising markers of myoepithelial cells [ 27 ].

MMP-2 activity, as a proteolytic enzyme, is necessary for cancer invasion and metastasis. All ECM elements can be cleaved by a specific MMPs but MMPs function is antagonized by their tissue inhibitors (TIMPs) [ 14 ]. The expression of MMP-2, MMP-9 and TIMP-1, 2, 3 has been evaluated in benign and malignant salivary gland tumors by Nagel *et al*. [ 15 ]. Their findings indicated significantly higher expression of MMP-2 in malignant tumors in comparison with benign neoplasms. In another study, Taher *et al*. [ 28 ] examined the MMP-2 expression in 22 MEC cases. However, they did not find any correlation between MMP-2 expression and histologic grade or tumor stage. On the contrary, we here observed significant positive correlation between MMP-2 expression and histologic grade in MEC. The difference in the results may be attributed to the smaller sample size in Taher *et al*.’s [ 28 ] study. In addition, Zheu *et al*. [ 16 ] investigated MMP-2 expression in 83 cases of ADCC. In accordance with our results, they observed no association between MMP-2 expression and prognostic indicators including histologic grade, lymph node metastasis and survival rate. These findings may be explained by the role of myoepithelial cells in regulating of ECM proteins and MMPs. In fact, some studies specified the necessity of ECM degradation as a prerequisite for neoplastic transformation of myoepithelial cells during carcinogenesis. Indeed, the MMP-2 and MMP-9 expressed by myoepithelial cells are usually inhibited by higher expression of TIMP-1 [ 2 , 18 ]. 

The correlation of MMP-2 and P63 expression has been addressed in oral squamous cell carcinoma. It is thought that P63 regulates MMPs production through variable mediators and pathways including repression of Wnt/B catenin response and snail-mediated epithelial-mesenchymal transition. In this respect, a peculiar role has been proposed for Δ Np63α in MMP-2 dependent invasiveness in SCC [ 29 ]. Importantly, our result also verified an association between MMP-2 and P63 expression in MEC samples. Nonetheless, further investigations are required to clarify the exact mechanism.

## Conclusion

In conclusion, our findings showed that MEC is devoid of myoepithelial cells. Accordingly, the difference in expression of P63 and maspin in ADCC and MEC highlighted the role and presence of myoepithelial cells in ADCC. The higher expression of P63 and maspin as the most promising myoepithelial markers in well-differentiated ADCCs suggests a tumor suppressor effect for myoepithelial cells. Considering the association between the evaluated markers and histologic grade as one of the prognostic indicators of ADCC and MEC, p63 in ADCC and MEC, maspin in ADCC and MMP-2 in MEC may be efficient predictors of clinical behavior.
